# A novel missense mutation (FGG c.1168G > T) in the gamma chain of fibrinogen causing congenital hypodysfibrinogenemia with bleeding phenotype

**DOI:** 10.1186/s41065-024-00308-0

**Published:** 2024-01-18

**Authors:** Nuo Xu, Liping Zheng, Zhehao Dai, Jun Zhu, Peng Xie, Shun Yang, Fei Chen

**Affiliations:** 1 Department of Spine Surgery, the Second Xiangya Hospital, Central South University, Changsha, China; 2 Department of Nephrology, Mengchao Hepatobiliary Hospital of Fujian Medical University, Fuzhou, China; 3 Department of Minimally Invasive Orthopedics, The Hunan University of Medicine General Hospital, Huaihua, China

**Keywords:** Congenital hypodysfibrinogenemia, Missense mutation, Synthesis, Secretion, Polymerization

## Abstract

**Background:**

Fibrinogen plays pivotal roles in multiple biological processes. Genetic mutation of the fibrinogen coding genes can result in congenital fibrinogen disorders (CFDs). We identified a novel heterozygous missense mutation, *FGG* c.1168G > T (NCBI NM_000509.6), and conducted expression studies and functional analyses to explore the influence on fibrinogen synthesis, secretion, and polymerization.

**Methods:**

Coagulation tests were performed on the patients to detect the fibrinogen concentration. Whole-exome sequencing (WES) and Sanger sequencing were employed to detect the novel mutation. Recombinant fibrinogen-producing Chinese hamster ovary (CHO) cell lines were built to examine the recombinant fibrinogen synthesis and secretion by western blotting and enzyme-linked immunosorbent assay (ELISA). The functional analysis of fibrinogen was performed by thrombin-catalyzed fibrin polymerization assay. In silico molecular analyses were carried out to elucidate the potential molecular mechanisms.

**Results:**

The clinical manifestations, medical history, and laboratory tests indicated the diagnosis of hypodysfibrinogenemia with bleeding phenotype in two patients. The WES and Sanger sequencing revealed that they shared the same heterozygous missense mutation, *FGG* c.1168G > T. In the expression studies and functional analysis, the missense mutation impaired the recombinant fibrinogen's synthesis, secretion, and polymerization. Furthermore, the in silico analyses indicated novel mutation led to the hydrogen bond substitution.

**Conclusion:**

The study highlighted that the novel heterozygous missense mutation, *FGG* c.1168G > T, would change the protein secondary structure, impair the “A: a” interaction, and consequently deteriorate the fibrinogen synthesis, secretion, and polymerization.

**Supplementary Information:**

The online version contains supplementary material available at 10.1186/s41065-024-00308-0.

## Background

Fibrinogen is a 340 kDa soluble plasma glycoprotein synthesized and secreted from the liver. It plays pivotal roles in multiple biological processes such as hemostasis, angiogenesis, and so on [1]. The fibrinogen polypeptide monomer consists of Aα, Bβ, and γ chains, encoded by *FGA*, *FGB*, and *FGG*, respectively [2]. After being linked by inter- and intra-chain disulfide bonds, the fibrinogen undergoes dimerization before being released into the bloodstream. Structurally, it exhibits a trinodular structure containing a central E-domain and two identical outer D-domains. The E-domain is formed by the N-terminus of the Aα, Bβ, and γ chains, and the D-domains are composed of the C-terminus of the Bβ and γ chains [3]. Notably, The γ chains within the D-domains (γD region) contain numerous functional sites and structures for fibrin polymerization, like “D:D” interface, γ-γ cross-linking, and high-affinity Ca^2+^-binding sites [4]. Therefore, the mutation occurring in the γD region may lead to potential deficiencies in fibrinogen quantity and quality.

Over 400 congenital fibrinogen disorders (CFDs) have been reported so far, and they showed different clinical features and molecular abnormalities [5]. The current classification system of CFDs relies on the functional and antigenic fibrinogen levels [6]. Hypofibrinogenemia or afibrinogenemia is characterized by low or absent plasma fibrinogen antigen levels. Dysfibrinogenemia or hypodysfibrinogenemia mainly displays reduced functional activity, possibly accompanied by qualitative fibrinogen deficiencies. Extensive studies have been conducted to explore the underlying mechanisms of fibrinogen disorders. It was revealed that genetic mutations occurring within the fibrinogen coding genes played crucial roles in the pathogenesis of CFDs, encompassing missense mutations, nonsense mutations, frame-shift mutations, splice-site abnormalities, and so on [7]. For one thing, mutations undermine DNA stability, mRNA splicing, and protein synthesis as well as the secretion of fibrinogen. For another, they adversely affect fibrinogen functions like fibrin polymerization or fibrinopeptide cleavage. So recognizing these mutations is significant for the diagnosis and prognosis of potential carriers.

We recently identified a novel heterozygous missense mutation, *FGG* c.1168G > T, in a 60-year-old female and her 30-year-old daughter with hypodysfibrinogenemia. Recombinant fibrinogen-producing CHO cell lines were established to evaluate the recombinant fibrinogen synthesis, secretion, and polymerization. In the present study, we aimed to explore and clarify the underlying genetic mechanism comprehensively.

## Results

### Clinical description

Patient 1 (propositus) was a 60-year-old woman admitted to the hospital because of a lumbar compression fracture. Before the percutaneous vertebroplasty, the blood clotting parameters showed severely low fibrinogen concentration. Notably, ecchymosis was observed in the lower back. This was uncommon in individuals without low plasma fibrinogen conditions and indicated bleeding risks. Upon inquiry about the medical history, she recalled significantly increased menstrual volume and prolonged duration before but did not receive treatment due to economic constraints several decades ago. During the surgery, she received a 200 mL fresh plasma infusion as a precautionary measure against unexpected surgical bleeding. Finally, the micro-invasive operation was successful and the bleeding (< 10 mL).

Patient 2 was a 30-year-old woman and the daughter of Patient 1. She had been experiencing abnormally heavy menstrual bleeding and causal moderate anemia for a long time. The low plasma fibrinogen concentration was detected by routine blood biochemical examination. The symptomatic treatment was ongoing, but the etiology remained unknown. The results of routine and special blood clotting tests were listed in Table 1. Notably, the ratios of Fg: C to Fg: Ag of these two patients were 0.42 and 0.66, which indicated the possibility of congenital hypodysfibrinogenemia.

**Table 1:** Routine and special coagulation

	Age	Clotting Times (s)			Fibrinogen (g/L)		FDP (ug/mL)
		PT	APPT	TT	Fg: C	Fg: Ag	
Patient 1	60	12.6	26.1	26.2	0.47	1.12	2.60
Patient 2	30	13.5	37.8	25.6	0.60	0.91	6.26
Healthy donor	28	11.1	25	15.4	3.52	3.10	1.40
Normal Range		10.0–14.0	24.8–34.6	14.0–21.0	2.00–4.00	2.00–4.00	0–5.00

*PT* Prothrombin time, *APTT* Activated partial thromboplastin time, *TT* Thrombin time, *Fg C* Fibrinogen activity, *Fg Ag* Fibrinogen antigen; Ratio: Fg: C/ Fg: Ag, *FDP* Fibrinogen degradation products

For the deceased status of Patient 1’s parents and husband many years ago, we were unable to obtain the clinical sample. Therefore, we could not explore the clinical significance of this mutation at the familial level.

### WES and Sanger sequencing

**Fig. 1:**
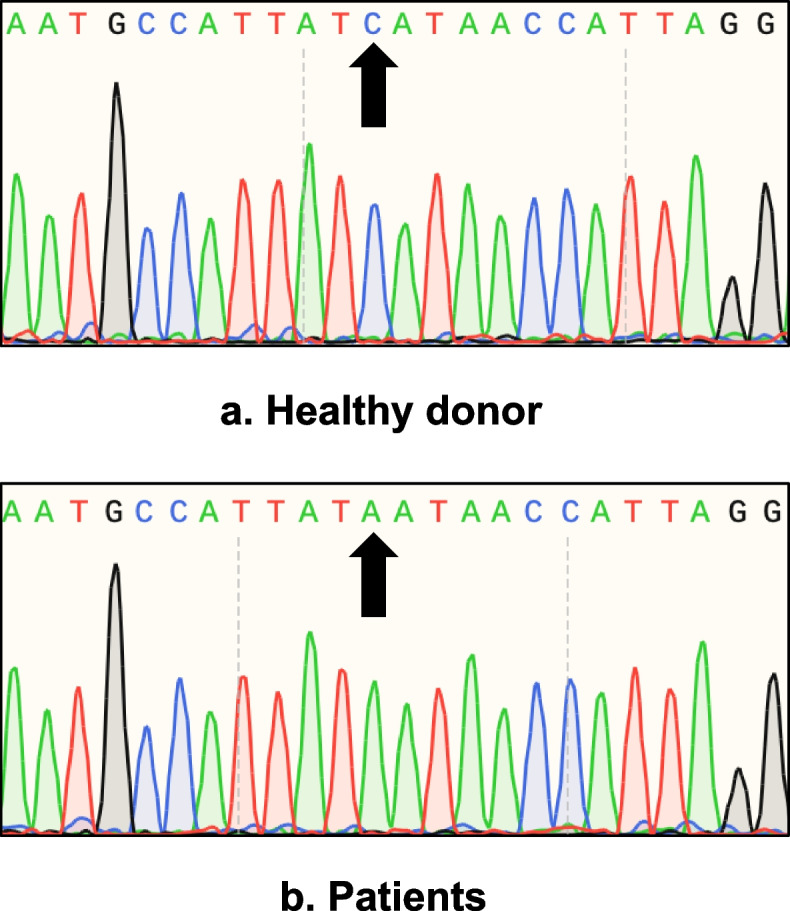
The Sanger sequencing results of the healthy donor (**a**) and patients(**b**) demonstrated that the single nucleotide appeared at position 1168 in the antisense strand of *FGG* (*FGG* c.1168G > T; NCBI NM_000509.6)

After careful consideration, we proposed the possibility of congenital hypodysfibrinogemia and performed the WES and Sanger sequencing on their blood samples. The WES results revealed a shared heterozygous nucleotide mutation at position 1168 in *FGG* exon 9 (*FGG* c.1168G > T), turning the aspartic acid into tyrosine at the 390th residue of the γ-chain (γD390Y, also called γD364Y in mature protein form). We predicted it to be a missense mutation by bioinformatic techniques, which had yet to be present in the GnomAD repository (reporting data on > 125,000 exomes and > 15,000 genomes; https://gnomad.broadinstitute.org/) and dnSNP database (https://www.ncbi.nlm.nih.gov/snp/). No genetic mutations were detected in *FGA* and *FGB*. Moreover, the results of Sanger sequencing on the *FGA*, *FGB,* and *FGG* were consistent with those of WES, which indicated the reliability of the WES (Fig. 1).

### Characterization of plasma fibrinogen

**Fig. 2:**
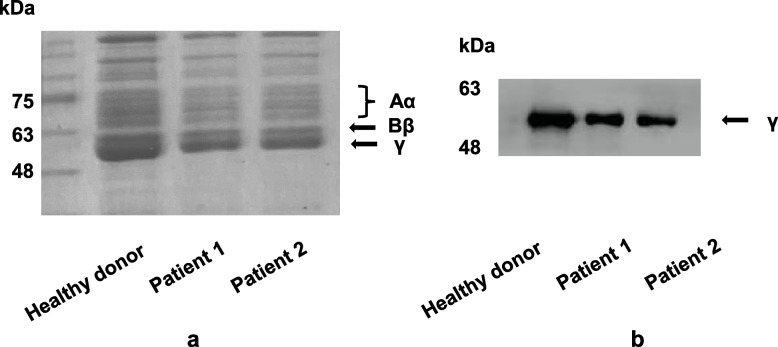
**a** By using SDS-PAGE to characterize the purified fibrinogens (3 μg), there were three bands compatible with the Aα, Bβ, and γ chains in the healthy donor and patients. **b** By using western blotting with anti-*FGG* polyclonal antibody, the single band position in the patients was consistent with that of the γ-chain from the healthy donor

On reducing conditions, there were three bands compatible with the Aα, Bβ, and γ chains in each sample, and no abnormal protein degradation was observed (Fig. 2a). By using western blotting, the single band position in the patients was consistent with that of the γ-chain from the healthy donor (Fig. 2b), supporting the findings from the WES and Sanger sequencing. Furthermore, it indicated that the mutation would not impair the synthesis of the fibrinogen Aα and Bβ chains. Therefore, we tended to explore the impact of the mutation on the γ-chain and fibrinogen synthesis.

### Synthesis and secretion of recombinant fibrinogens in CHO cell lines

**Fig. 3:**
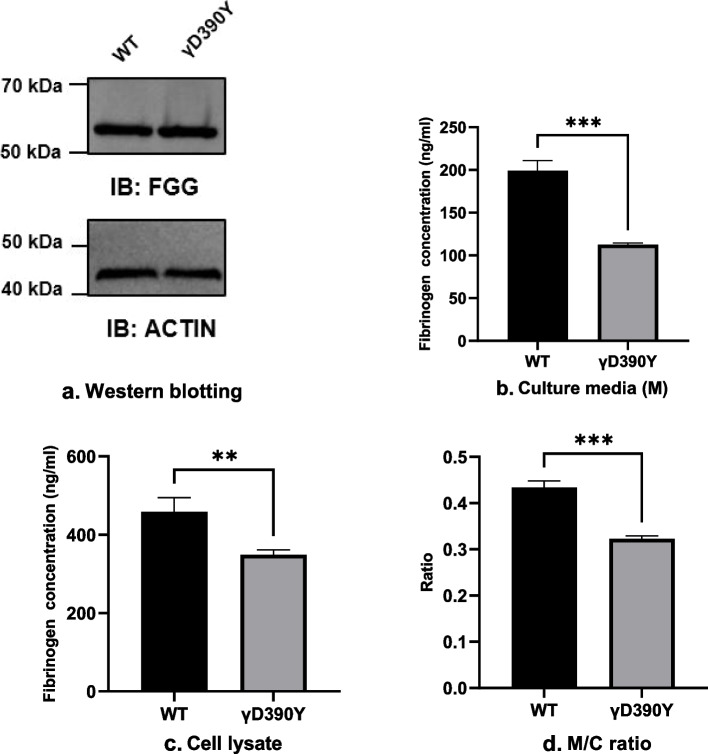
**a** Western blotting analysis for fibrinogen γ-chain from recombinant WT and γD390Y fibrinogen-producing CHO cell lines. **b** The fibrinogen concentration of culture media and cell lysates.** c** The fibrinogen concentration of cell lysates. **d** The ratio of fibrinogen concentration in culture media to that in cell lysates. The fibrinogen concentrations were detected by ELISA. **, *P* < 0.01; ***, *P* < 0.001

To investigate the impact of the missense mutation on the γ-chain and fibrinogen expression, we established stable recombinant WT and γD390Y γ-chain-producing CHO cell lines. We first evaluated the synthesis of the fibrinogen γ-chain by western blotting analysis. As expected, it was shown that the molecular weight and expression of the γD390Y γ-chain were the same as that of the WT. The results indicated the mutation did not reduce the γ-chain expression, which was accordant with the previous bioinformatic prediction (Fig. 3a).

Next, we co-transfected the *FGA* and *FGB* expression vectors into the recombinant WT and γD390Y γ-chain producing CHO cell lines to generate recombinant fibrinogen and evaluated the impact of missense mutation on fibrinogen synthesis and secretion. The results demonstrated that fibrinogen concentrations in the cell lysates from the recombinant WT and γD390Y fibrinogen-producing CHO cell lines were 459.10 ± 20.72 ng/mL and 349.10 ± 7.21 ng/mL, respectively. Fibrinogen concentrations in culture media from the recombinant WT and γD390Y fibrinogen-producing CHO cell lines were 199.0 ± 12.60 ng/mL and 112.6 ± 1.22 ng/mL. The fibrinogen concentration ratios of the culture media to cell lysates of the recombinant WT and γD390Y fibrinogen-producing CHO cell lines were 0.43 ± 0.0081 and 0.32 ± 0.0032, respectively (Fig. 3b-d). Taken together, the results showed that the missense mutation in *FGG* significantly impaired fibrinogen synthesis and secretion.

### Thrombin-catalyzed fibrin polymerization

**Fig. 4:**
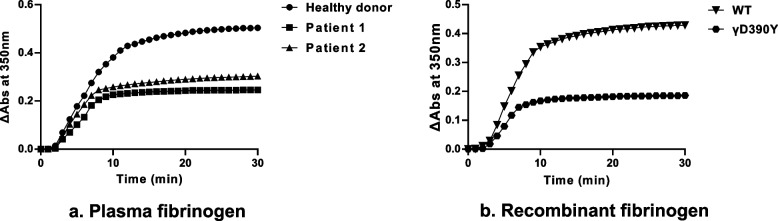
Thrombin-catalyzed plasma fibrinogen **a** and recombinant fibrinogen polymerization (**b**). The fibrinogen (0.5 mg/mL) was initiated with thrombin (0.05U/mL). The experiments were performed in triplicate and representative polymerization curves were indicated

The turbidity curves of the plasma fibrinogen and recombinant fibrinogen were depicted in Fig. 4, and three related parameters were presented in Supplementary Table 1. The results demonstrated a significant impairment in the fibrin polymerization ability of the patient-derived plasma fibrinogen compared to that from the healthy donor. Similarly, the recombinant γD390Y fibrinogen showed significantly lower fibrin polymerization ability than the recombinant WT fibrinogen.

### In silico molecular analysis

**Fig. 5:**
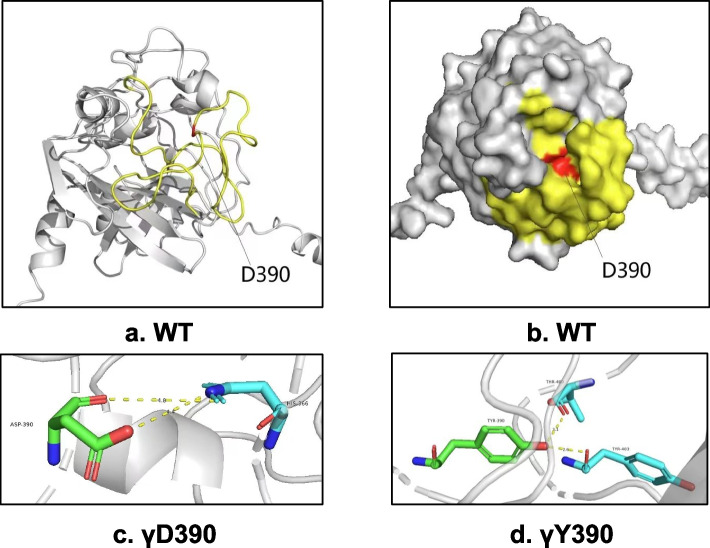
**a**, **b** Predicted tertiary structure and surface structure of the WT γ-chain, respectively. The yellow area indicated the hole “a”. Red referred to the γD390. **c**, **d** The HB between the γ390 and other residues in WT and γD390Y fibrinogen γ-chain. The green and blue sticks referred to different amino acids. The yellow dotted lines meant HB between residues. Hole “a” is a constitutive complementary-binding pocket in the γD region that would interact with the polymerization site termed knob “A”. HB, hydrogen bond

Protein modeling has emerged as a powerful technique to speculate and elucidate in-depth molecular mechanisms underlying the quantitative and qualitative defects in fibrinogen. We performed an in silico molecular analysis to evaluate the impact on the secondary structure of fibrinogen caused by each amino acid substitution. Compared to the γ chain of the WT fibrinogen, the amino acid alternation induced transformations in hydrogen bond (HB). The HB between γD390 and γHistidine366 (γH366) was replaced by those between γD390 and γThreonine400 (γT400), as well as γD390 and γD403 (Fig. 5). By reviewing relevant studies, we discovered the variants γD390N, γD390H, and γD390V led to dysfibrinogenemia, characterized by polymerization defects without low fibirnogen concentration [8]. These seems contradicted from the current study, so additional analysis were needed to figure out the differences among the variants. Compared with the wild type, these three mutations only influenced the quality and quantity of HB between the D390 and H366, which cause exceptional polymerization defects (Supplementary Fig. 1). However, D390Y caused new hydrogen bond formation with another amino acid, which was believed to induce significant and different changes.

## Discussion

We identified a new heterozygous missense mutation, *FGG* c.1168G > T, in two patients leading to congenital hypodysfibrinogenemia with bleeding phenotype. Initially, the coagulation tests on the propositus showed a decreased fibrinogen concentration level and reduced fibrinogen activity/antigen ratio, indicative of hypodysfibrinogenemia. Subsequently, we observed a similar condition in her daughter and proposed a genetic predisposition. Therefore, the WES and Sanger sequencing was performed, and the results showed the same single nucleotide mutation. Since no gene abnormalities in *FGA* and *FGB* genes, we focused on exploring the impact of the novel missense mutation in *FGG* on fibrinogen synthesis, secretion, and polymerization.

Admittedly, there are some similarities between hypodysfibrinogenemia and hypofibrinogenemia [9]. For instance, they can be asymptomatic and discovered by accident during routine coagulation tests in certain cases. Additionally, the bleeding tendency can be triggered under stimuli like trauma, surgery, and pregnancy [10]. However, the reduction of functional and antigenic fibrinogen levels in hypodysfibrinogenemia was disproportional. Some researchers took the ratio of Fg: C to Fg: Ag below 0.7 as an efficient standard to distinguish them [11]. Nevertheless, others challenged the reliability due to the variations in reference materials and assay reproducibility [12]. Hence, in the current study, expression studies and functional analysis were necessary to acquire an accurate diagnosis and determine the adverse effects of the variants. Moreover, the thrombotic risk in hypodysfibrinogenemia can significantly increase relative to hypofibrinogenemia, and special caution is required in performing plasma replacement therapy [13]. Thus, we performed limited plasma infusion under close monitoring of the coagulation in this case.

It was well established that the fibrin polymerization would begin once the thrombin eliminated the N-terminal fibrinopeptide A (FPA) in the fibrinogen α chain [14]. The cleavage of the FPA exposed a polymerization site termed knob “A” to a constitutive complementary-binding pocket (hole “a”) in the γD region and promoted their combination [15]. The “A: a” interaction would cause the fibrin monomers to align in a staggered overlapping end-to-middle domain arrangement and consequently form double-stranded twisting fibrils. Remarkably, the γ337-379 (in mature protein form) were identified as the primary fibrinogen polymerization site. Because this domain played a critical role in the electrostatic interactions that facilitated the important first step in fibrin polymerizatio, where the mutations would have significant detrimental effects [16]. In the present study, the γD390Y was located at this region and resulted in impaired polymerization ability, which was consistent with previous reports. Interestingly, the novel mutation showed a different clinical manifestation of low fibrinogen concertration besides polymerization defect. According to the in silico analyses, γD390Y led to the more significant changes in HB than other variants. As HB serves as a strong inter-molecular force and plays pivotal roles in the formation, stabilization, and function of protein, γD390Y is enough to significantly impair the structural stability, synthesis, and secretion of the fibrinogen and consequently lead to more severe clinical manifestations.

## Conclusion

In conclusion, the current study revealed that the novel heterozygous missense mutation, *FGG* c.1168G > T, would change the protein secondary structure, impair the “A: a” interaction, and consequently deteriorate the fibrinogen synthesis, secretion, and polymerization.

## Materials and methods

### Coagulation test

Peripheral blood samples from patients were collected into standard anti-coagulant tubes. After centrifugation at 3000 rpm for 10 min, the platelet-poor plasma was obtained and then used for coagulation tests within 2 h. Subsequently, the prothrombin time (PT), activated partial thromboplastin time (APTT), thrombin time (TT), and fibrinogen activity (Fg: C) were measured by the Clauss method with STA-R Evolution automatic analyzer (Diagnostic Stago, Inc). The fibrinogen antigen (Fg: Ag) and fibrinogen degradation products (FDP) were assayed by immunoturbidimetry using the automatic analyzer (Beckman Coulter, Inc). The fibrinogen activity/antigen ratio cutoff of 0.7 was considered diagnostic for dysfibrinogenemia, which has not been validated so far.

### DNA isolation, WES, and Sanger sequencing

The DNA from both patients for genetic analysis was isolated from peripheral blood by using a genome DNA isolation kit (Qiagen, Hiden, Germany), according to the manufacturer’s instructions. Afterward, WES and Sanger sequencing were carried out by Kingmed Center for Clinical Laboratory (Changsha, China).

### Purification and characterization of fibrinogen

We performed immunoaffinity chromatography to purify the plasma fibrinogen from the patients and healthy donors with anti-IF-1 monoclonal antibody (LSI Medience) conjugated to a Sepharose 4B column. Furthermore, ammonium sulfate precipitation methods were utilized to purify the fibrinogen from the recombinant fibrinogen-producing CHO cell lines [17, 18]. The acquired fibrinogen was resuspended and the concentration was measured by a BCA protein assay kit (Beyotime Biotech, P0010S).

Subsequently, we applied the sodium dodecyl sulfate–polyacrylamide (SDS-PAGE) to analyze the purity and characterization of the purified fibrinogen in reducing conditions (10% polyacrylamide gel) and stained it with Coomassie Brilliant Blue G-250.

### Construction of mini-gene expression vectors

The *FGA* (NM_000508.5), *FGB* (NM_001184741.1), and *FGG* (NCBI NM_000509.6) (both wild type and mutant type) cDNAs were synthesized by Tsingke Biotech. Then, the *FGA* and *FGB* cDNAs were cloned at XhoI and BamHI sites of the pcDNA 3.1-3xFlag vector, and *FGG* cDNA was cloned at XbaI and BamHI sites of the pCDH-CMV-MCS-EF1-puromycin vector. The primers (Supplementary Table 2) were designed by the SnapGene 6.0.2 software (GSL Biotech LLC).

### Lentivirus packaging, infection, and recombinant fibrinogen γ chain-producing cell lines establishment

The HEK293T cells were seeded in 6-well culture plates and transfected with recombinant, lentiviral, and packaging vectors, including pMDL, VSVG, and REV at a ratio of 10:5:3:2 using Lipofectamine 2000. The virus was collected, filtered, and added to the CHO cells 48 h later, followed by medium replacement 12 h later. Afterward, the 4ug/ml puromycin was added to screen out the stable recombinant wild type (WT) and mutant type (MT) fibrinogen γ chain-producing CHO cell lines. In addition, the *FGA* and *FGB* expression vectors were transfected into the stable cell lines to produce the recombinant fibrinogen.

### Western blotting and ELISA

After the transfection, both the recombinant WT and MT fibrinogen-producing CHO cells were lysed in lysis buffer (50 mM Tris–HCl (pH 7.4), 150 mM NaCl, 1 mM EDTA and 1% Triton X-100) containing protease inhibitor cocktail (Sigma, P2714-1BTL) on ice for 30 min followed by centrifugation. Protein concentration was measured by the BCA method, as mentioned before. Soluble lysates were subjected to SDS-PAGE and transferred to polyvinylidene fluoride (PVDF) membranes (Merck Millipore). After blocking with 5% BSA (BioFroxx, Germany) or fat-free milk, membranes were probed with primary antibodies (Proteintech, 15,841–1-AP) at 4 ℃ overnight and secondary anti-rabbit (ABclonal, AS063) or anti-mouse (ABclonal, AS064) antibodies at room temperature for 1 h. Signals were visualized after incubation with Clarity Western ECL substrate (Bio-Rad, Hercules, CA, USA). The ELISA (Abcam, ab241383) was performed to detect fibrinogen concentration in the cell lysates and culture media under the manufacturer’s instructions.

### Thrombin-catalyzed fibrin polymerization

The turbidity curves of fibrin polymerization were recorded at 350 nm using a UV-1280 (Shimadzu, Japan). Human α-thrombin (Yeasen, China)-catalyzed fibrin polymerization was performed, as described before. We aimed to acquire three parameters: lag time, maximum slop (Max-slope), and absorbance change (ΔAbs) in 30 min.

### In silico molecular analysis

After acquiring the protein sequence, we performed protein structure homology modeling with the Swiss-model platform (https://swissmodel.expasy.org/) on the recombinant WT and MT fibrinogen gamma chain. Then mutation was analyzed by Mutagenesis Wizard of PyMOL. We selected each rotamer with the least steric clashes of available rotamers during the process.

### Statistical analysis

All Data were presented as the mean ± SD. Student’s t-tests were performed to figure out the differences among groups using GraphPad Prism 9.0. A statistical significance was considered when *P* < 0.05.

### Supplementary Information

**Additional file 1: Supplementary Figure 1. **(a) -(c) The HB between the different variants of γ390 and other residues in the fibrinogen γ chain. The green and blue sticks referred to different amino acids. The yellow dotted lines meant HB between residues. HB, hydrogen bond. N, Asparagine. H, Histidine. V, Valine.

**Additional file 2: Supplementary Table 1. **Thrombin-catalyzed fibrin polymerization.

**Additional file 3: Supplementary Table 2. **

## Data Availability

The data that support the findings of this study are not openly available due to reasons of sensitivity and are available from the corresponding author upon reasonable request.

## Abbreviations

CFDs: Congenital fibrinogen disorders
WES: Whole-exome sequencing
CHO: Chinese hamster ovary
ELISA: Enzyme-linked immunosorbent assay
γD region: γ Chains in the D-domains
HB: Hydrogen bond
γH366: γHistidine366
γT400: γThreonine400
N: Asparagine
H: Histidine
V: Valine
FPA: Fibrinopeptide A
PT: Prothrombin time
APTT: Activated partial thromboplastin time
TT: Thrombin time
Fg: C: Fibrinogen activity
Fg: Ag: Fibrinogen antigen
FDP: Fibrinogen degradation products
SDS-PAGE: Sodium dodecyl sulfate–polyacrylamide
WT: Wild type
MT: Mutant type
PVDF: Polyvinylidene fluoride
Δabs: Absorbance change
