# Supplementary material for: A novel missense mutation (FGG c.1168G > T) in the gamma chain of fibrinogen causing congenital hypodysfibrinogenemia with bleeding phenotype
Source: Hereditas. 2024 Jan 18;161:4. doi: 10.1186/s41065-024-00308-0 (PMC10795222; doi:10.1186/s41065-024-00308-0)
Supplement: Supplementary file 1 — Additional file 1: Supplementary Figure 1. (a) -(c) The HB between the different variants of γ390 and other residues in the fibrinogen γ chain. The green and blue sticks referred to different amino acids. The yellow dotted lines meant HB between residues. HB, hydrogen bond. N, Asparagine. H, Histidine. V, Valine. [file 41065_2024_308_MOESM1_ESM.pdf]

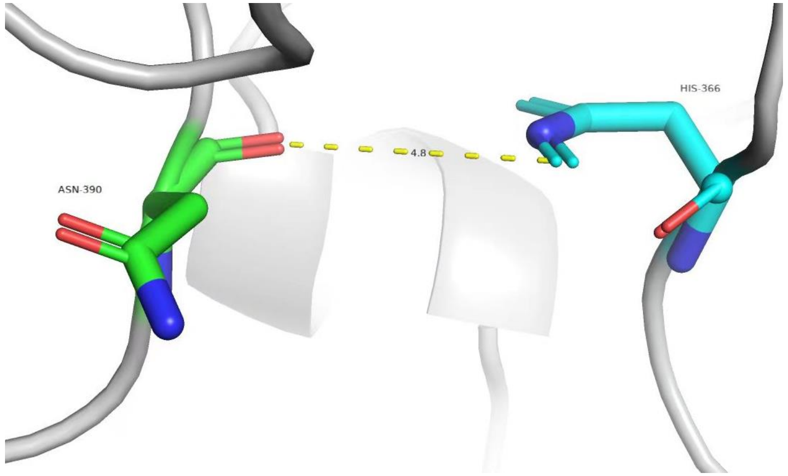

a. γN390

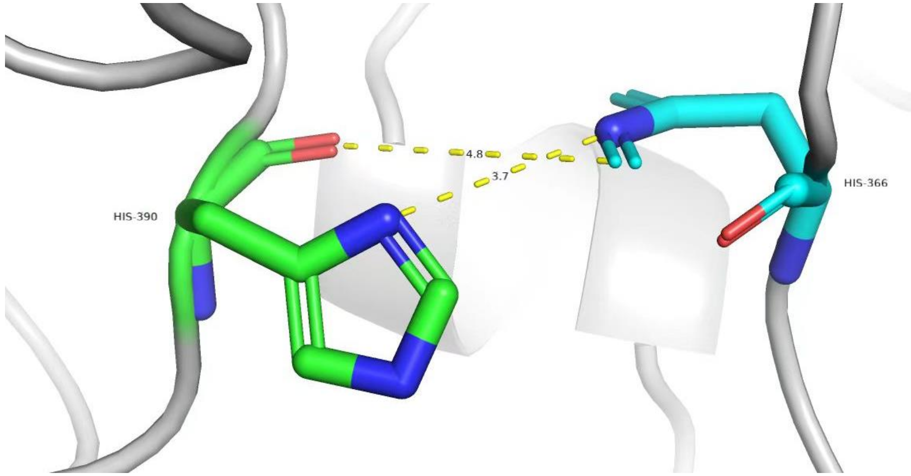

b. γH390

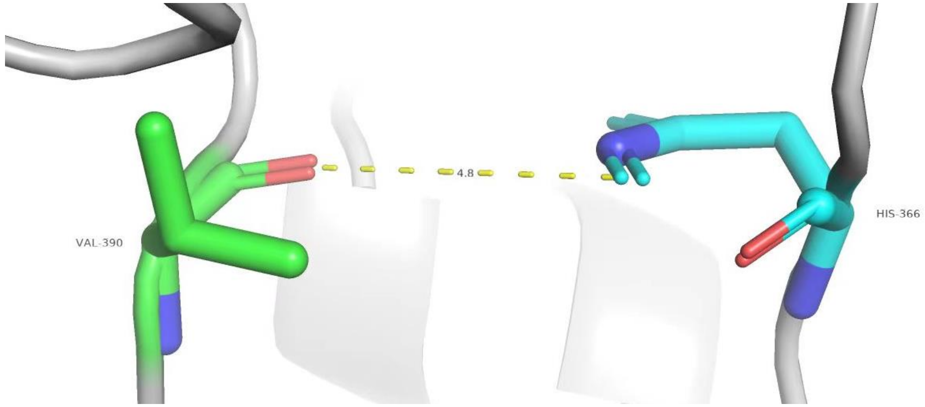

c. γV390

Supplementary Figure 1. (a) - (c) The HB between the different variants of γ390 and other residues in the fibrinogen γ chain. The green and blue sticks referred to different amino acids. The yellow dotted lines meant HB between residues. HB, hydrogen bond. N, Asparagine. H, Histidine. V, Valine.
