# Supplementary material for: A novel missense mutation (FGG c.1168G > T) in the gamma chain of fibrinogen causing congenital hypodysfibrinogenemia with bleeding phenotype
Source: Hereditas. 2024 Jan 18;161:4. doi: 10.1186/s41065-024-00308-0 (PMC10795222; doi:10.1186/s41065-024-00308-0)
Supplement: Supplementary file 2 — Additional file 2: Supplementary Table 1. Thrombin-catalyzed fibrin polymerization. [file 41065_2024_308_MOESM2_ESM.docx]

**Supplementary Table 1. Thrombin-catalyzed fibrin polymerization**

|  | **Thrombin-catalyzed fibrin polymerization** | | | | | |
| --- | --- | --- | --- | --- | --- | --- |
|  | **Lag time (min)** | | **Max-slope (×10^-3^/s)** | | **ΔAbs (30 min)** | |
| **Plasma fibrinogen** | | | | | | |
| **Healthy donor** | 1.5 ± 0.1 |  | 0.92 ± 0.27 |  | 0.504 ± 0.020 |  |
| **Patient 1** | 2.4 ± 0.3 | *** | 0.67 ± 0.13 | NS | 0.246 ± 0.012 | *** |
| **Patient 2** | 2.2 ± 0.3 | *** | 0.77 ± 0.18 | NS | 0.303 ± 0.015 | *** |
| **Recombinant fibrinogen** | | | | | | |
| **WT** | 1.8 ± 0.2 |  | 0.99 ± 0.21 |  | 0.429 ± 0.019 |  |
| **γD390Y** | 2.3 ± 0.1 | * | 0.59 ± 0.15 | NS | 0.186 ± 0.007 | *** |

Footnote: The results were presented as mean ± SD. WT, wild type; *: Compared with healthy donor or wild type, *p*<0.05; **: Compared with healthy donor or wild type, *p*<0.01; ***: Compared with healthy donor or wild type, *p*<0.001. NS, not significant.
