# Supplementary material for: A novel missense mutation (FGG c.1168G > T) in the gamma chain of fibrinogen causing congenital hypodysfibrinogenemia with bleeding phenotype
Source: Hereditas. 2024 Jan 18;161:4. doi: 10.1186/s41065-024-00308-0 (PMC10795222; doi:10.1186/s41065-024-00308-0)
Supplement: Supplementary file 3 — Additional file 3: Supplementary Table 2. [file 41065_2024_308_MOESM3_ESM.docx]

**Supplementary Table 1.**

| **Gene** | **Primer sequence (5’- 3’)** |  |
| --- | --- | --- |
| FGA | F: TTGGTACCGAGCTCGGATCCATGTTTTCCATGAGGATCGT |  |
|  | R: AAGGGCCCTCTAGACTCGAGACTTAGTCTAGGGGGACAGG |  |
| FGB | F: TTGGTACCGAGCTCGGATCCATGAAAAGGATGGTTTCTTGGAGC |  |
|  | R: AAGGGCCCTCTAGACTCGAGTTGCTGTGGGAAGAAGGG |  |
| FGG | F: ACCTCCATAGAAGATTCTAGAATGAGTTGGTCCTTGCACCC |  |
|  | R: ATCCTTCGCGGCCGCGGATCCTTAAACGTCTCCAGCCTGTT |  |
